# Is Varicocele Truly Unilateral? Contralateral Involvement and Bilateral Testicular Effects in Male Infertility: A Narrative Review

**DOI:** 10.3390/jpm16080417

**Published:** 2026-08-04

**Authors:** Aris Kaltsas, Andreas Koumenis, Evangelos N. Symeonidis, Fotios Dimitriadis, Nikolaos Sofikitis

**Affiliations:** 1 Third Department of Urology, Attikon University Hospital, School of Medicine, National and Kapodistrian University of Athens, 12462 Athens, Greece; akaltsas@med.uoa.gr (A.K.); a_koumeni@icloud.com (A.K.); 2 Department of Urology II, European Interbalkan Medical Center, 55535 Thessaloniki, Greece; evansimeonidis@gmail.com; 3 Department of Urology, Faculty of Medicine, School of Health Sciences, Aristotle University of Thessaloniki, 54124 Thessaloniki, Greece; helabio@yahoo.gr; 4 Laboratory of Spermatology, Department of Urology, Faculty of Medicine, School of Health Sciences, University of Ioannina, 45110 Ioannina, Greece

**Keywords:** varicocele, bilateral varicocele, subclinical varicocele, laterality, color Doppler ultrasonography, male infertility, nutcracker phenomenon, venous reflux, varicocelectomy, internal spermatic vein

## Abstract

**Background/Objectives:** Varicocele is usually clinically left-sided, but imaging and mechanistic studies suggest that contralateral venous reflux or bilateral testicular effects may be more common than physical examination indicates. This narrative review critically examines whether varicocele-associated infertility is better conceptualized as an asymmetric disorder with potential bilateral anatomical or functional involvement and considers the implications for diagnosis and treatment. **Methods:** PubMed/MEDLINE and Scopus were searched from inception to 21 June 2026 for studies on varicocele laterality, bilateral and subclinical disease, color Doppler ultrasonography, venography, and unilateral versus bilateral repair. Reference lists and contemporary clinical guidelines were also reviewed. Because the included studies differed substantially in patient selection, age, operator technique, diagnostic criteria, and reference standards, reported bilateral rates were not interpreted as head-to-head estimates of diagnostic sensitivity or as pooled prevalence estimates. **Results:** Physical examination identifies predominantly left-sided clinical disease. Bilateral involvement is reported more frequently when both sides are assessed using Doppler ultrasonography or venography, but estimates vary widely across referral-enriched and highly selected cohorts. Human and experimental evidence supports several mechanisms by which a clinically unilateral lesion may have bilateral functional effects, including shared scrotal hyperthermia, oxidative stress, endocrine disturbance, and venous cross-communication. Bilateral repair is consistent with standard treatment criteria when both varicoceles are clinically palpable. In men with a clinical left varicocele and non-palpable right-sided reflux, comparative evidence is mixed, and current guidelines do not support routine treatment of imaging-only disease. **Conclusions:** Varicocele is best viewed as an asymmetric disorder that may be anatomically or functionally bilateral in selected men, rather than as a universally bilateral disease. Deliberate bilateral clinical assessment and standardized bilateral ultrasonography when imaging is clinically indicated may improve phenotyping and operative planning. However, contralateral imaging abnormalities should not automatically be converted into a surgical indication.

## 1. Introduction

Varicocele—abnormal dilatation and tortuosity of the pampiniform plexus secondary to retrograde venous flow in the internal spermatic (gonadal) vein—is the most common surgically correctable cause of male subfertility [1,2]. It is detected in a sizeable proportion of men evaluated for infertility and is associated with impaired semen parameters, sperm DNA damage, and, in a subset of men, reduced testosterone production [2,3]. Repair can improve semen parameters and pregnancy rates in appropriately selected men, although the magnitude of benefit and the optimal indications remain debated across professional societies [4,5].

For more than a century, varicocele has been taught as a predominantly left-sided disease, classically described in roughly 85–90% of cases as unilateral left, with bilateral disease in approximately 10% and isolated right disease in well under 1% on clinical examination [1]. The anatomical rationale is familiar: the left internal spermatic vein is longer and drains into the left renal vein at a near-perpendicular angle, whereas the right drains obliquely into the inferior vena cava (IVC); the left renal vein is additionally susceptible to extrinsic compression between the superior mesenteric artery and the aorta (the “nutcracker” phenomenon) [1,6,7,8,9]. In cohorts assembled by clinical examination, the left side dominates, bilateral disease is reported in a minority, and isolated right-sided varicocele is distinctly uncommon [10].

Although one healthy testis can often sustain fertility, this observation does not establish that varicocele-associated infertility necessarily reflects bilateral testicular failure. Total sperm output represents the combined contribution of both testes, and marked dysfunction of one testis may be sufficient to impair semen quality. In addition, pregnancy is a couple-level outcome influenced by female age, ovarian reserve, and other reproductive factors. Nevertheless, clinically unilateral disease may coexist with non-palpable contralateral reflux or may produce bilateral functional effects through shared thermal, vascular, endocrine, or oxidative pathways. These two possibilities—anatomical bilateral involvement and bilateral functional consequences—should be examined separately.

The purpose of this narrative review is therefore to evaluate, rather than presuppose, whether varicocele is best regarded as an asymmetric disorder with potential bilateral consequences. Four questions are addressed: (1) which anatomical and hemodynamic factors govern laterality; (2) how study population and method of ascertainment influence the reported frequency of bilateral involvement; (3) which mechanisms may connect a clinically unilateral lesion with bilateral testicular effects; and (4) under which clinical phenotypes, if any, bilaterality should alter management. Throughout the review, anatomical involvement, functional impairment, and the indication for contralateral treatment are considered as related but distinct concepts.

## 2. Literature Search and Scope

This work is a narrative—not a systematic—review and does not follow PRISMA methodology. PubMed/MEDLINE and Scopus were searched from database inception to 21 June 2026, combining the terms varicocele, bilateral, subclinical, contralateral, laterality, right, color Doppler ultrasonography, nutcracker, and varicocelectomy, supplemented by hand-searching of reference lists and of contemporary guideline and consensus documents; recent meta-analyses and guideline updates published between 2024 and 2026 were specifically sought. No language restriction was applied, and non-English studies were included where they contributed unique data.

The scope is deliberately confined to the laterality of varicocele in the context of male infertility—its anatomical and hemodynamic basis, how the diagnostic method influences the reported prevalence of bilateral involvement, the mechanisms linking the two testes, and the clinical, surgical, and guideline implications; varicocele in children and adolescents, pain-predominant disease, and comparative embolization technique are considered only where they bear on laterality. Priority was given to randomized trials, meta-analyses, and prospectively designed clinical series, with mechanistic studies included to support biological plausibility; single-group and selected-population data—central to several bilaterality claims—are identified as such throughout. As a purposive rather than exhaustive synthesis, selection bias is possible, and no quantitative pooling or formal risk-of-bias assessment was undertaken.

**Methodological limitations of the evidence base.** The diagnostic modalities discussed in this review were generally evaluated in different clinical populations and were rarely compared head-to-head in consecutively enrolled patients with blinded interpretation. Accordingly, differences in reported bilateral rates were treated as differences in ascertainment under heterogeneous conditions, rather than as direct estimates of comparative sensitivity or specificity. Spectrum bias, referral bias, verification bias, operator experience, patient age, examination position, Doppler thresholds, and the systematic or non-systematic assessment of the contralateral side were considered when interpreting individual studies. No quantitative pooling or formal risk-of-bias assessment was performed.

## 3. The Anatomical and Hemodynamic Basis of Laterality

The laterality of varicocele is grounded in the asymmetric anatomy and hemodynamics of gonadal venous drainage. This section examines the structural and pressure-related factors that account for left-sided predominance, while clarifying why they establish a gradient of susceptibility rather than a categorically left-only disease.

### 3.1. Asymmetry of the Internal Spermatic Venous Drainage

The structural asymmetry of gonadal venous drainage is the foundation of varicocele laterality. The left internal spermatic vein typically ascends to terminate in the left renal vein at an approximately right-angled junction, whereas the right internal spermatic vein usually drains directly and obliquely into the IVC below the right renal vein. The left venous column is longer than the right. In a standing column of blood, hydrostatic pressure is determined by column height rather than by vessel caliber; a longer, more vertically oriented left column therefore transmits greater hydrostatic load to the pampiniform plexus when valvular competence is lost [11,12]. These features account for the earlier and more pronounced clinical expression of left-sided disease without, in themselves, excluding right-sided involvement.

### 3.2. Valvular Incompetence and the Nutcracker Phenomenon

Retrograde flow requires failure of the antireflux mechanism—absent, congenitally incompetent, or acquired-incompetent valves in the internal spermatic vein—permitting transmission of renal venous pressure to the scrotal veins. Superimposed on this is the nutcracker phenomenon: compression of the left renal vein in the aortomesenteric angle, which raises left renal venous pressure and promotes gonadal vein reflux and collateralization [6,7,8]. In a sonographic case–control study, nutcracker findings were substantially more frequent among men with varicocele than controls, and intrinsic anatomical differences in the aortomesenteric and hilar segments of the left renal vein were proposed as direct contributors to varicocele onset [9]. The nutcracker phenomenon is predominantly a left-sided mechanism; its relevance here is twofold—it reinforces left predominance, and, when severe or symptomatic, it identifies a secondary, mechanistically distinct cause of varicocele that may warrant dedicated evaluation.

### 3.3. Why the Left Predominates—Without Excluding the Right

The anatomical arguments above explain a gradient of susceptibility, not a categorical left-only disease. The right internal spermatic vein is also valved and also drains a vertical venous column; right-sided reflux is mechanically possible and, when the right vein drains anomalously into the right renal vein rather than the IVC, may be facilitated. Crucially, right-sided reflux of lower magnitude may remain subclinical—below the threshold of palpable detection—while still being demonstrable on imaging or venography [13]. The central interpretive point of this review follows directly: a disease that is bilateral in its venous substrate but asymmetric in its hemodynamic severity will appear unilateral to any diagnostic test whose sensitivity is calibrated to the more severe side.

### 3.4. Venous Collaterals and Retroperitoneal Cross-Communication

The internal spermatic venous systems are not isolated conduits. They form networks with collateral and bypass channels—including cremasteric (external spermatic), gubernacular, and deferential veins—and communicate across the midline through retroperitoneal anastomoses. In the contemporary surgical literature, incomplete control of exactly these collateral and duplicated channels is a dominant driver of varicocele persistence and recurrence, underscoring how anatomically complex and interconnected the drainage really is [14]. Two implications bear on laterality. First, the presence of crossover channels provides an anatomical route by which reflux arising on one side could influence the contralateral venous bed. Second, selected venographic series report a high frequency of collaterals and retroperitoneal bypasses bilaterally, which the investigators interpret as evidence that the disease is intrinsically a bilateral network disorder rather than a focal left-sided lesion [11,15].

## 4. Detecting Bilateral Involvement: Ascertainment, Heterogeneity, and Methodological Limitations

Any estimate of bilateral varicocele involvement must specify both the population studied and the method of ascertainment. The available literature does not permit a valid universal ranking of diagnostic sensitivity because physical examination, ultrasonography, thermography, and venography were generally applied to different clinical populations and under different protocols. The following sections therefore describe how ascertainment influences the observed bilateral rate, rather than presenting these modalities as directly comparable diagnostic tests.

### 4.1. Clinical Grading Versus Subclinical Disease

The widely used Dubin–Amelar clinical grading system [16] distinguishes grade I (palpable only during Valsalva), grade II (palpable at rest), and grade III (visible through the scrotal skin), with subclinical varicocele defined as disease not palpable on examination but demonstrable by imaging. By construction, subclinical disease is invisible to palpation. Because right-sided varicoceles are disproportionately of lower grade, a substantial fraction of right-sided disease falls into the subclinical category and is therefore systematically missed by examination-based ascertainment [13,17].

### 4.2. Physical Examination: Clinical Role and Limitations

Physical examination performed in a warm room with the patient standing and during Valsalva remains the basis of clinical diagnosis and determines eligibility for treatment under current infertility guidelines. Its performance, however, is influenced by examiner experience, body habitus, room conditions, and the grade of the lesion. A contemporary diagnostic-accuracy study reported moderate overall accuracy of physical examination compared with color Doppler ultrasonography, with better performance among experienced andrologists and urologists [18]. The consequence for laterality estimates is direct and historically documented. As early as 1986, scrotal ultrasonography in 50 infertile men reclassified disease that palpation had called unilateral: a left varicocele was confirmed in all clinically affected men and detected by sonography in 12 additional men, raising left-sided disease to 34/50 (68%)—of whom 12/50 (24%) had a subclinical (impalpable) left varicocele. A right varicocele was demonstrated in 24/50 (48%) and was never found in isolation, always accompanying a left lesion, so that 24 of the 34 men with any sonographic varicocele (70%) had bilateral disease [19]. In a large mixed epidemiological series, the diagnosis of bilateral varicocele likewise rose markedly when detailed ultrasonographic evaluation replaced physical examination alone—from roughly one-seventh to about one-third of varicocele patients—and clinically silent right-sided involvement was present in the great majority of bilateral cases [20]. The simplest reading is that palpation does not so much measure laterality as measure the more severe side.

### 4.3. Color Doppler Ultrasonography: Criteria and Concordance

Color Doppler ultrasonography (CDU) is a non-invasive method that can detect subclinical disease by measuring pampiniform venous diameter and demonstrating retrograde flow, typically augmented by the Valsalva maneuver [3]. Reported diameter thresholds for clinical varicocele cluster around a maximum venous diameter of roughly 2.45 mm at rest and 2.95 mm during Valsalva in one prospective series [21], with other groups finding resting diameter the most discriminating single parameter and reflux duration providing additional diagnostic information [22]. Importantly, CDU and clinical grading are only moderately concordant: ultrasonographic grades corresponding to lower thresholds map onto clinically subclinical disease, so that several ultrasonographically detectable varicoceles remain impalpable on examination [23]. CDU thus reclassifies a meaningful share of apparently unilateral (left-only) clinical varicoceles as bilateral once the contralateral side is imaged. A persistent limitation is the absence of a single, internationally standardized CDU definition, which constrains cross-study comparison and itself limits cross-study comparability and precise estimation of bilateral involvement [3,23].

Recent clinical series reinforce this point. In a prospective case series of 329 infertile men, repeat expert color Doppler plus intraoperative Doppler reclassified bilaterality to 98.5% despite roughly one-third of cases having been initially referred as unilateral, with repeat ultrasonography closely matching the intraoperative contralateral venous diameter [24]. In a separate prospective Doppler cohort, bilateral varicocele was identified in a substantial proportion of clinically diagnosed cases [25]. These series are selected and should not be read as population prevalence estimates, but they reinforce the methodological claim that laterality depends strongly on how actively the contralateral side is interrogated.

When ultrasonography is clinically indicated, it should be performed using a standardized bilateral protocol rather than as a diameter-only examination. Grey-scale, color, and spectral Doppler assessment should be performed on both sides in the supine and standing positions, at rest and during Valsalva, documenting the largest venous diameter, reflux duration, testicular volume, and the anatomical level and pattern of reflux. Reflux lasting more than 2 s while standing during Valsalva is generally considered abnormal; however, neither venous diameter nor reflux duration has been validated as an isolated threshold for contralateral surgery [26,27]. Doppler assessment may also assist surgical planning by identifying refluxing collateral or deferential venous pathways: in a historical pediatric and adolescent series of 449 patients undergoing laparoscopic varicocelectomy, the addition of inguinal color Doppler ultrasonography to detect deferential reflux was associated with a reduction in persistence or recurrence from 13.7% to 1%, although the retrospective, historical-control, pediatric design limits direct extrapolation to adult infertility practice [28,29].

### 4.4. Thermography and Venography

Spermatic venography is the most direct demonstration of reflux and culprit venous channels and retains a role in complex or previously treated cases, where it can map duplicated veins and collaterals and guide targeted embolization [14]. It is also the basis of the strongest bilaterality claims in the literature: in venographic and thermographic series of infertile men, the proportion classified as bilateral is far higher than in palpation- or ultrasound-based cohorts (Section 4.5) [11,15]. In one prospective study, contact thermography, Doppler sonography, and venography raised the detection of right-sided varicocele from 8.2% on palpation to between 74% and 84%, yielding bilateral disease in roughly four-fifths of patients [15]. Venography is invasive and not appropriate as a screening tool; its findings, while mechanistically informative, are drawn from selected populations and should be interpreted with that caveat.

### 4.5. Method Dependence and the Epidemiology of Bilateral Involvement

The relationship between detection method and apparent laterality is summarized in Table 1. The general direction is consistent across the literature: more intensive and systematic contralateral assessment is generally associated with higher reported bilateral proportions; however, the magnitude of this association cannot be separated reliably from differences in population selection and study protocol.

The methodological argument does not, by itself, establish that varicocele *is* bilateral; it establishes that the unilateral picture is partly an artifact of insensitive ascertainment, and that any valid prevalence estimate must be method-qualified.

Reported bilateral proportions vary widely, from low estimates in population-based clinical screening to very high rates in selected imaging, surgical, or venographic cohorts. These differences cannot be attributed solely to the diagnostic modality; they also reflect age, infertility status, referral enrichment, whether the contralateral side was examined systematically, patient position, Doppler criteria, operator experience, and the selective use of invasive reference standards (Table 2). In examination-based cohorts, the left side dominates and bilateral disease is a minority finding; in one large clinical cohort of men diagnosed with varicocele, the distribution was approximately four-fifths left-only, with bilateral and isolated-right disease each comprising a smaller fraction [10]. Notably, isolated right-sided disease in this imaged referral cohort (~8%) far exceeds the classic clinical estimate of well under 1%, a gap best explained by ascertainment—liberal cross-sectional imaging in a referral population detects right-sided involvement that unselected palpation misses—rather than by a true difference in prevalence. When ultrasonography is applied systematically, bilateral disease is identified in a substantial proportion—on the order of a third or more of varicocele patients in some series, and up to ~70% of infertile men with varicocele in early sonographic work [19,20]. At the extreme, thermographic and venographic series from Gat and colleagues report bilateral disease in roughly 80–84% of infertile men, with collaterals and retroperitoneal bypasses present bilaterally in the majority; in their flagship cohort, among 255 men with confirmed varicocele the disease was left-only in 17.6%, right-only in 1.5%, and bilateral in 80.8%, and approximately 90% of the right-sided lesions in bilateral cases were subclinical [11,15]. These figures are the empirical core of the strong “bilateral disease” hypothesis and are addressed critically in Section 5 and Section 8.

Contemporary imaging-focused cohorts extend this spectrum. One case series reported an extreme late-ascertainment estimate of 98.5% bilaterality after expert reassessment of 329 infertile men [24]; a smaller prospective Doppler cohort identified bilateral disease in a substantial proportion of clinically diagnosed varicocele cases [25]; and a 2026 Doppler hemodynamic cohort of men with primary infertility likewise found bilateral involvement in almost all Doppler-diagnosed cases [30] (its hemodynamic correlates of semen quality are considered in Section 6.1). These cohorts are heterogeneous and referral-enriched, but they provide contemporary support for the same direction of effect shown in older sonographic and venographic series.

Two further epidemiological observations are relevant. First, bilateral (and right-sided) involvement appears to increase with age: prevalence has been reported to rise by roughly 10% per decade, reaching high frequencies in older men, consistent with progressive valvular failure over time [31], and bilateral subclinical disease is more frequent in older than in younger men with subclinical varicocele [17]. Second, the population studied matters. Infertility-clinic cohorts and adolescents systematically imaged for a clinical left varicocele are enriched for contralateral findings, with reported right-sided rates up to ~40% when subclinical disease is included. Such cohorts should not be compared directly with unselected screening populations [1,20]. This is expected if bilateral disease carries greater functional consequence (Section 6).

**Table 2:** Selected studies reporting bilateral or contralateral varicocele involvement according to population and ascertainment method. Estimates vary widely because of differences in population selection, age, infertility status, definition of clinical versus subclinical varicocele, patient position, Doppler criteria, use of venography, and whether the contralateral side was systematically assessed; the reported figures are therefore illustrative of diagnostic method-dependence and should not be pooled.

Study (Ref)	Population	Ascertainment	Reported Bilateral Involvement	Note
**Infertility cohorts—imaging or venography**				
Trum et al. 1996 [32]	63 infertile men	Palpation + contact thermography + color Doppler; spermatic venography as reference	≈10% bilateral (3/31 venography-positive men)	Reference-standard venographic cohort gave a low bilateral rate—definitions and method matter (counterpoint)
McClure & Hricak 1986 [19]	50 infertile men	Palpation versus scrotal ultrasonography	24/34 ultrasound-detected varicoceles (70%) bilateral	12/50 (24%) had a subclinical left varicocele; right reflux never isolated, always accompanying left
Trussell et al. 2003 [33]	58 infertility-clinic men with clinical varicocele	Scrotal ultrasonography after clinical diagnosis	45/58 (77.5%) bilateral on ultrasound	Routine ultrasound revealed occult contralateral disease; of 13 non-bilateral men, 8 left-only, 1 right-only, and 4 had no varicocele on ultrasound
Gat et al. 2004 [15]	286 infertile men (255 with varicocele)	Thermography + Doppler + venography	206/255 (80.8%) bilateral	~90% of right-sided lesions subclinical
Mulyadi et al. 2026 [30]	40 men with primary infertility and Doppler-diagnosed varicocele	Testicular-artery Doppler + grading	38/40 (95%) bilateral (1 right-only, 1 left-only)	Highly selected Doppler-diagnosed cohort
Almekaty et al. 2023 [24]	329 infertile men	Repeat expert CDU + intraoperative Doppler	Initially referred bilateral in 229/329 (69.6%); bilateral documented in 324/329 (98.5%) after repeat expert CDU + intraoperative Doppler	Selected surgical/infertility cohort; not a population prevalence
**Infertility cohorts—clinical ± Doppler**				
Misra & Srivastava 2025 [25]	45 infertile men with clinical varicocele; 45 controls	Clinical examination + scrotal Doppler	37.8% bilateral (55.6% left, 6.7% right) among 45 cases	Prospective single-center cohort
**General-population/screening cohorts**				
Damsgaard et al. 2016 [34]	7035 healthy young men, six European countries	Standardized physical examination	~1.1% overall (79/7035); ~7% of varicocele-positive men	Large general-population comparator; clinical examination gives a low estimate
Pfeiffer et al. 2006 [35]	2756 children + 2008 adolescents (Hamburg)	Genital examination + Doppler	Bilateral 1.2% (children), 7.2% (adolescents)	Screening population; much lower absolute bilaterality than infertility cohorts
**Age-specific or special populations**				
Elmer DeWitt et al. 2018 [10]	4060 men with varicocele (clinical referral)	Examination ± imaging	Left 3258 (80.2%), isolated right 337 (8.3%), bilateral 465 (11.5%) of 4060	Right-varicocele men older, higher BMI, more cross-sectional imaging
Chen 2017 [17]	Men with subclinical varicocele	Color Doppler ultrasonography	Bilateral subclinical more frequent in older men (≈33% versus ≈10%)	Age-related increase

Abbreviations: CDU, color Doppler ultrasonography; US, ultrasonography; BMI, body mass index; IVC, inferior vena cava.

## 5. Pathophysiological Coupling of the Two Testes

**Figure 1:**
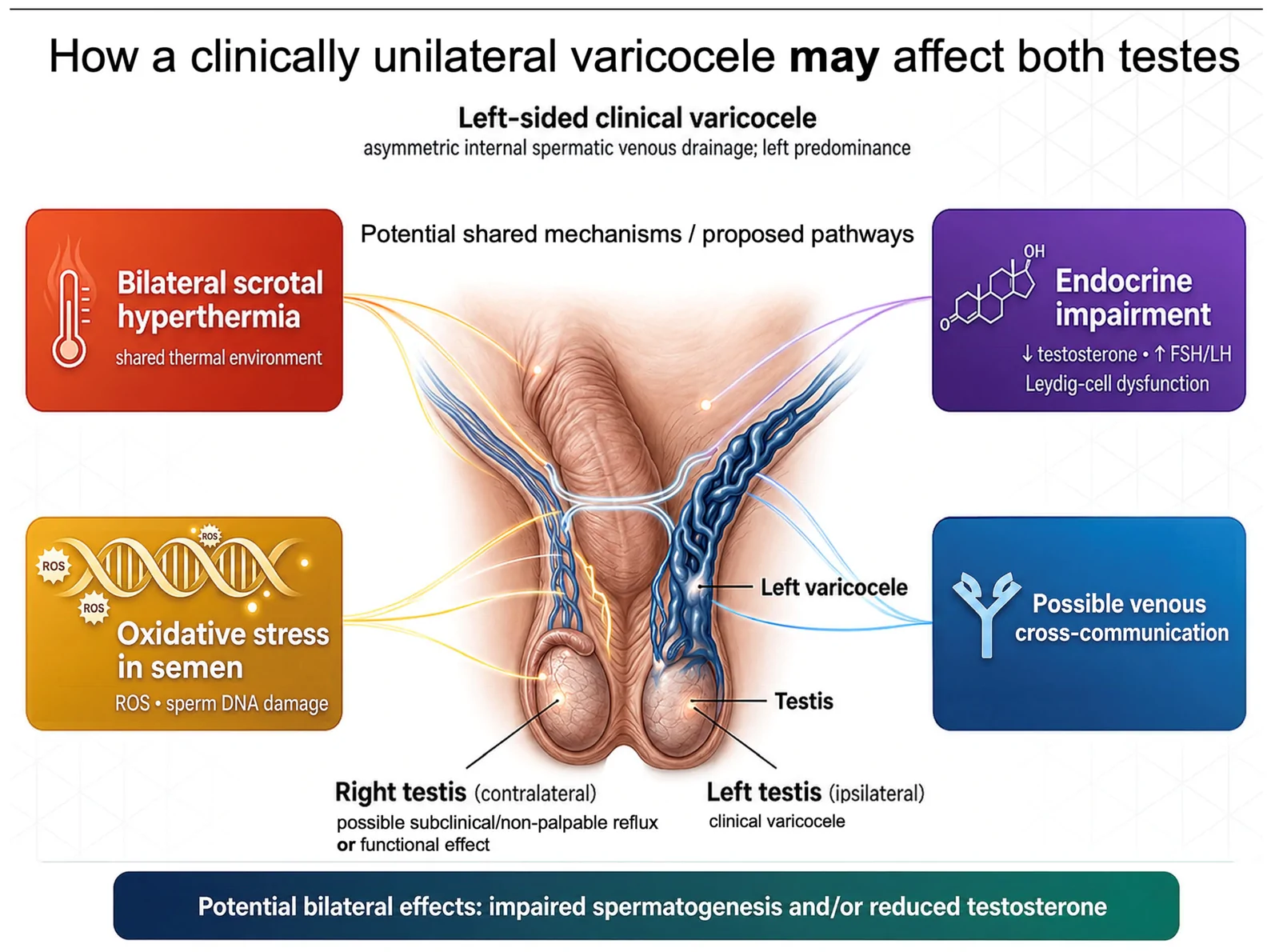
Conceptual pathways through which a clinically dominant left-sided varicocele may be associated with bilateral testicular effects. Human evidence is strongest for a shared increase in scrotal temperature; oxidative and endocrine abnormalities are associated with varicocele but do not identify the anatomical side of origin, whereas cross-midline venous effects remain mechanistically plausible but incompletely validated. The figure depicts potential pathways and should not be interpreted as evidence that every clinically unilateral varicocele causes bilateral testicular injury. FSH, follicle-stimulating hormone; LH, luteinizing hormone; ROS, reactive oxygen species.

If varicocele can be unilateral yet produce bilateral testicular dysfunction, the mechanisms linking the two testes require explanation (Figure 1). Several—anatomical, thermal, oxidative, and humoral—are plausible and partly evidenced.

### 5.1. Why Clinically Unilateral Disease May Have Bilateral Effects

The observation that a single healthy testis can often support fertility does not prove that varicocele-associated infertility is necessarily bilateral. Total sperm output reflects the combined contribution of both testes, and severe unilateral dysfunction may reduce the ejaculate below clinically relevant thresholds. In addition, infertility is a couple-level outcome influenced by female reproductive factors. Nevertheless, human thermal studies [36], experimental models [37,38,39,40], and the frequent detection of contralateral reflux [19,20] support the possibility that the contralateral testis may also be affected—either because the right side harbors its own, often non-palpable, venous abnormality or because the clinically dominant left-sided lesion produces remote functional effects. These mechanisms are not mutually exclusive.

### 5.2. The Hydrostatic-Pressure/Hypoxia (Gat–Gornish) Hypothesis

Gat and colleagues proposed, on the basis of venography and fluid-mechanics modeling, that destruction of internal spermatic vein valves generates vertical blood columns and pathological hydrostatic pressure in the testicular microcirculation, ultimately exceeding arteriolar pressure and reversing the normal arteriovenous gradient. The proposed consequence is persistent testicular hypoxia—affecting both the seminiferous epithelium and the Leydig cell compartment, with a corresponding fall in testosterone—and the model predicts that the disease is intrinsically bilateral, expressed earlier and more severely on the left because of the longer venous column [11,12,31]. This framework is internally coherent and influential, but it must be reported as a *hypothesis*: the supporting venographic and histological data derive substantially from a single group, in selected infertile populations undergoing intervention, and have not been independently replicated at comparable scale. It is presented here as the strongest articulation of the bilateral-disease thesis, not as consensus physiology.

### 5.3. Shared Mechanisms Acting Bilaterally

Independent of the hydrostatic model, several mechanisms can plausibly transmit injury from a unilateral lesion to the contralateral testis.

Bilateral scrotal hyperthermia is one such route: the two testes share a scrotal thermal environment maintained below core temperature by countercurrent heat exchange, and venous pooling impairs this system. Direct intraoperative thermistor measurements in infertile men demonstrated that intratesticular and scrotal surface temperatures are elevated in varicocele and—critically—that a unilateral varicocele is associated with bilateral elevation of scrotal surface temperature [36]; this is among the strongest pieces of human evidence that a one-sided lesion exerts a two-sided effect. Elevated scrotal temperature has, in turn, been linked to subfertility in men with subclinical varicocele [17].

Oxidative stress is a further mediator: varicocele is a recognized cause of oxidative stress in semen and sperm DNA damage [2,41]. In men with bilateral varicocele, semen-based markers shift toward a pro-oxidant state, with reduced antioxidant capacity and increased reactive oxygen species (ROS), accompanied by activation of regulated cell-death pathways (ferroptosis, pyroptosis, necroptosis) in spermatozoa [42]. Oxidative stress measured in semen, however, reflects the combined contribution of spermatozoa, the testes, epididymides, leukocytes, and accessory glands; it characterizes the overall biological state of the ejaculate but cannot identify whether the injury originated from the left testis, the right testis, or both. Similarly, sperm DNA fragmentation is a whole-ejaculate biomarker and should not be interpreted as direct evidence that a contralateral imaging abnormality is clinically causal.

A 2026 systematic review and meta-analysis of sperm DNA fragmentation (SDF) in varicocele (40 eligible studies; 27 contributing extractable SDF data to the pooled comparison) found significantly higher SDF in men with varicocele than in controls (mean difference 15.34 percentage points; 95% confidence interval (CI) 9.74–20.90; *p* < 0.001), providing a contemporary quantitative basis for incorporating SDF into selected evaluations [43]. Consistent with a reversible oxidative component, a 2024 systematic review and meta-analysis (29 studies, 1491 infertile men with clinical varicocele) reported a significant post-repair reduction in sperm DNA fragmentation (standardized mean difference −1.13) and in seminal malondialdehyde, an oxidative-stress marker [44].

A prospective microsurgical varicocelectomy cohort further suggested that a high preoperative DNA-fragmentation index may carry predictive value, with a reported threshold predicting postoperative semen improvement, indicating that sperm-DNA integrity may help identify biologically active and potentially reversible varicocele-associated injury [45].

Reflux of renal and adrenal metabolites and altered hemodynamics may also contribute: retrograde flow can expose the testis to renal and adrenal venous constituents, and intratesticular arterial hemodynamics are measurably altered. In subclinical varicocele, abnormal intratesticular resistive and pulsatility indices—rather than venous caliber alone—distinguish men with impaired semen quality, and bilateral disease is over-represented in that group [46].

Finally, cross-midline venous communication offers an anatomical conduit: retroperitoneal anastomoses (Section 3.4) provide a route by which reflux originating on one side could raise pressure in the contralateral venous bed [11].

Endocrine and testicular-volume signals point in the same direction: a 2026 systematic review and meta-analysis found that men with varicocele had higher FSH and LH, lower total testosterone, smaller testicular volumes, and worse semen parameters than men without varicocele, supporting combined spermatogenic and Leydig-cell impairment rather than a purely semen-level phenotype [47]. This endocrine impairment appears partly reversible: a 2025 systematic review and meta-analysis found that varicocele repair improves testicular endocrine function, with a rise in serum testosterone most evident in men with low baseline levels [48].

### 5.4. Experimental Evidence of Contralateral Injury

Animal models support bilateral functional consequences of a unilateral lesion. In a rat model, experimental left varicocele produced progressive histological damage in both testes over time, with the most severe (including Sertoli-cell-only) changes ipsilaterally, indicating that a unilateral venous lesion can compromise the contralateral gonad [37]. Experimentally induced left varicocele has likewise been associated with bilateral elevation of intratesticular temperature and testicular blood flow in rats and dogs [38] and with sustained, bilateral suppression of intratesticular testosterone accompanied by reduced testicular steroidogenic-enzyme activity [49]; contemporary rat models reproduce the fall in testosterone together with Leydig-cell stress and germ-cell apoptosis in the varicocele testis [50]. Notably, these bilateral blood-flow and temperature changes persist after ipsilateral orchidectomy, indicating that the contralateral response does not depend on the presence of the ipsilateral testis [39,40]. These are direct experimental demonstrations of a contralateral effect, but they derive from animal models; interspecies extrapolation requires caution, and they establish biological plausibility rather than proof of the same mechanism in men.

### 5.5. Distinct Molecular Signatures of Unilateral Versus Bilateral Disease

Comparative sperm-proteomic analysis suggests that unilateral and bilateral varicocele may differ qualitatively, not only in degree. In preliminary, largely single-cohort work, bilateral disease has been associated with a distinct pattern of differentially expressed and post-translationally modified (acetylated) spermatozoal proteins, with dysregulation of the proteasome complex and pathways linked to mitochondrial dysfunction, oxidative stress, and apoptosis—suggesting a more severe and qualitatively different molecular phenotype in bilateral disease [51]. Histopathological prognostic work in bilateral subclinical varicocele similarly indicates measurable, side-specific testicular changes that carry prognostic information for post-operative semen recovery [52]. These data are consistent with bilaterality being biologically meaningful rather than incidental, but they derive from small, largely single-center cohorts and require independent validation.

### 5.6. Discordance Between Varicocele Grade and Testicular Dysfunction

A recurring and clinically pivotal observation is that the size or clinical grade of a varicocele correlates poorly with the degree of testicular dysfunction. Early sonographic work emphasized that the deleterious effect of varicocele on spermatogenesis is largely independent of its size [19], and adolescent semen-analysis series have likewise found semen parameters to be independent of varicocele grade, with the testicular-volume differential—rather than grade—predicting impaired sperm concentration and motility [53]. Histological data are less consistent, as some biopsy series report grade-related increases in testicular oxidative damage [54,55]; grade–dysfunction discordance is therefore better established for semen parameters rather than for histological outcomes. This decoupling of grade from function has been reinforced by a 2025 cohort in which clinical grade did not correlate with sperm-DNA-fragmentation severity nor with the magnitude of DNA-fragmentation improvement after repair [56]. The implication for laterality is important: a low-grade or subclinical right-sided varicocele cannot be assumed to be functionally trivial simply because it is small, and may contribute to bilateral testicular impairment to a degree disproportionate to its palpable size. This decoupling of grade from function is part of the rationale for not dismissing the contralateral side on the basis of low grade alone (Section 6).

## 6. Clinical Significance: Does Bilaterality Matter for Outcomes?

The practical question is whether recognizing bilaterality changes prognosis or treatment. The evidence suggests it carries prognostic weight and can influence surgical strategy, though key questions remain unresolved.

### 6.1. Bilateral Subclinical Disease and Semen Quality

Several lines of evidence indicate that bilateral involvement is associated with worse or deteriorating semen parameters. In men with subclinical varicocele, bilateral disease and abnormal intratesticular hemodynamics—not maximum venous diameter or reflux grade—best distinguished those with abnormal semen analysis [46]. Beyond Doppler indices, dynamic contrast-enhanced magnetic resonance imaging (MRI) has shown that testicular perfusion—notably the wash-in rate—differs in infertile men with clinical varicocele, providing a functional imaging correlate of impaired testicular function [57]. In young men with bilateral subclinical varicocele and asthenozoospermia, total motile sperm count identified individuals at risk of measurable semen deterioration over follow-up, with the lowest baseline counts at highest risk [58]. Predictive modeling in subclinical varicocele likewise associates bilateral disease, smaller testicular volume, higher scrotal temperature, and adverse intratesticular indices with subfertility [17], and histopathological indices in bilateral subclinical disease carry prognostic value for semen recovery after microsurgical correction [52]. The consistent signal is that bilateral subclinical disease is not benign by default and warrants surveillance.

Contemporary Doppler hemodynamic data are concordant: in a 2026 primary-infertility cohort with Doppler-diagnosed varicocele, right testicular end-diastolic velocity correlated with sperm concentration and motility and remained an independent predictor of sperm concentration, suggesting that arterial-inflow/downstream-resistance metrics may help identify clinically consequential involvement [30].

### 6.2. Bilateral Versus Unilateral Varicocelectomy

The available evidence should be interpreted separately for bilateral palpable disease and for a clinical left varicocele accompanied only by imaging-detected right reflux; these phenotypes are not interchangeable. In infertile men with bilateral palpable varicoceles and abnormal semen parameters, bilateral repair is consistent with standard treatment criteria, and comparative cohorts generally favor treatment of both clinically affected sides [59,60]. By contrast, evidence for adding right-sided repair when the right lesion is non-palpable is mixed: randomized data are conflicting. Sun et al. (*n* = 358) [61] reported significantly greater improvements in semen parameters and a higher spontaneous pregnancy rate after bilateral repair, whereas the randomized study of Zheng et al. (*n* = 104) [62] found no benefit of bilateral over left-only repair in semen parameters, reproductive hormones, testicular volume, or pregnancy; meta-analyses favor bilateral repair on average but pool heterogeneous definitions of subclinical disease, surgical approaches, and follow-up [63,64]. The evidence therefore does not justify a routine bilateral strategy for imaging-only right reflux.

Where disease is genuinely bilateral, both observational and randomized data favor bilateral repair over unilateral repair in infertile men (Table 3). In men with a larger left and a small but palpable right varicocele, bilateral microsurgical repair produced substantially greater improvement in motile sperm concentration than left-only repair (≈+96% versus ≈+43%), leading the authors to conclude that even a small unrepaired palpable right varicocele continues to harm bilateral testicular function [59]. In a larger comparative series, bilateral repair again outperformed unilateral repair for motility (≈+8% versus ≈+4%) and spontaneous pregnancy (49% versus 36%), a pattern interpreted as a detrimental “dose-effect” of varicocele burden [60]. Two meta-analyses concur: bilateral varicocelectomy was associated with higher spontaneous pregnancy rates than unilateral repair (odds ratio ≈ 1.9) with greater motility gains [63], and, in the specific scenario of left clinical plus right subclinical disease, randomized data favored bilateral repair for progressive motility, morphology, and pregnancy (odds ratio ≈ 1.7), although sperm concentration differences were not significant [64]. The largest individual randomized trial in left clinical plus right subclinical varicocele (358 men) reported significantly greater improvements in concentration, morphology, and progressive motility and a higher spontaneous pregnancy rate after bilateral than after unilateral microsurgical repair (42.5% versus 26.0%) [61]; an earlier prospective comparative study in men with oligoasthenozoospermia reached the same conclusion and recommended concomitant repair of the right subclinical lesion [65]. Not all trials concur—at least one randomized study found no advantage to bilateral repair, but it was confounded by marked baseline left testicular hypotrophy, which may signify established, irreversible damage and illustrates the central importance of patient selection [1]; a separate randomized trial (*n* = 104) similarly found no benefit of bilateral over left-only repair [62]. Collectively, the evidence supports bilateral repair when both lesions are clinically palpable, but does not establish routine contralateral surgery when the right-sided abnormality is detectable only by imaging.

**Clinical indications for bilateral repair.** No validated objective threshold currently identifies which imaging-only right-sided varicocele should be repaired. In adult infertility practice, bilateral repair is supported when both varicoceles are clinically palpable and the man is attempting conception, has abnormal semen parameters, and otherwise meets established criteria for treatment after consideration of the female partner and the couple’s reproductive timeline. A right-sided lesion detected solely by imaging is not an independent surgical indication. Reproducibility of reflux, right testicular volume, semen characteristics, sperm DNA fragmentation, reproductive hormones, previous treatment, and couple-level factors may inform counseling or trial eligibility, but should be regarded as clinical modifiers rather than guideline-endorsed indications [4,5].

**Pediatric and adolescent considerations.** Adult infertility data should not be extrapolated directly to children and adolescents. Current European Association of Urology (EAU) pediatric guidance recommends offering surgery primarily when a persistent testicular size difference greater than 2 mL or 20% is documented; symptomatic disease, an additional contralateral testicular condition, bilateral palpable varicocele, abnormal semen parameters in an older adolescent, or substantial scrotal swelling may also justify treatment on an individualized basis. Clinical grade alone, including grade III disease, is not a sufficient indication. These criteria should be discussed separately from adult couple-based infertility indications [66].

**Choice of surgical approach in bilateral disease.** Bilaterality influences operative planning but does not determine a single preferred technique. Laparoscopy permits treatment of both gonadal veins through the same intraperitoneal access and may be operationally efficient for bilateral repair, particularly in pediatric and adolescent practice. In adult infertile men, microsurgical inguinal or subinguinal varicocelectomy is generally favored because magnification facilitates preservation of the testicular artery and lymphatics and is associated with lower recurrence and hydrocele rates than non-microsurgical approaches. The bilateral-access advantage of laparoscopy should therefore be balanced against patient age, venous anatomy, the need for lymphatic sparing, previous surgery, and local expertise [67,68].

Recent outcome literature also refines technique rather than laterality per se. A 2026 systematic review and meta-analysis of intraoperative Doppler during microsurgical subinguinal varicocelectomy reported effects on arterial preservation and the number of veins ligated, but marked heterogeneity and few studies limited firm conclusions about reproductive benefit [69]. This supports anatomy-informed completeness of venous control, particularly in complex or bilateral repair, while not resolving the laterality question.

### 6.3. The Contralateral Subclinical Varicocele Controversy

Two distinct clinical questions should be separated. The first is whether an isolated non-palpable varicocele should be repaired; here the evidence is largely negative. The second is whether a non-palpable right-sided lesion should be treated at the same time as a guideline-eligible clinical left varicocele; here the evidence is mixed and does not currently support routine contralateral treatment. The first is whether correcting a subclinical varicocele *in isolation* benefits fertility; here the evidence is largely negative. A randomized trial of isolated subclinical varicocelectomy found higher sperm density and total motile sperm count after high ligation, but no pregnancy advantage over observation; pregnancy was 6.7% versus 10.0%, surgery versus observation [70], and a systematic review and meta-analysis of 13 studies (1357 men) concluded that correction of subclinical varicocele produces only a minor, clinically insignificant improvement in semen parameters and no increase in pregnancy, against a background of high bias and no standardized definition [71]. Consistent with this, a prospective multicenter study comparing microsurgical ligation with observation in men with isolated subclinical varicocele found no significant improvement in sperm concentration or progressive motility and only a modest gain in morphology, reinforcing that isolated subclinical repair confers limited fertility benefit [72]. The second question is whether adding repair of a subclinical right varicocele to repair of a clinical left varicocele improves outcomes; here the randomized and observational data reviewed in Section 6.2 are more favorable [59,61,65]. The reconciliation is that the contralateral subclinical lesion appears to matter chiefly as an *additive* burden on top of clinical disease rather than as a stand-alone target. The practical corollary is that contralateral subclinical repair is defensible in selected men—particularly when accompanying a clinical left varicocele in an infertile couple—but is not supported as an isolated intervention, and patient selection, rather than routine, non-selective contralateral treatment, is where the evidence points [4,5].

A small prospective randomized study has, however, reported higher sperm count and a higher natural-pregnancy rate after microsurgical treatment than conservative management in men with subclinical varicocele and impaired semen parameters [73]. Because this trial was small and single-center, its signal should be treated as hypothesis-generating rather than practice-changing.

### 6.4. Bilateral Disease in Non-Obstructive Azoospermia

The relevance of bilaterality extends to severe phenotypes. Bilateral varicocele is reported in men with azoospermia, including cases with normal follicle-stimulating hormone in whom the varicocele is a plausible contributor [74]. Whether to repair varicocele in this setting remains debated, because reported outcomes are inconsistent [75,76]. More broadly, varicocele repair in men with non-obstructive azoospermia (NOA) and clinical varicocele increases the odds of sperm appearance in the ejaculate and of surgical sperm retrieval, and improves clinical pregnancy rates with intracytoplasmic sperm injection (ICSI) in controlled studies [75]; in a surgical cohort, varicocele grade and favorable testicular histopathology (hypospermatogenesis or late maturation arrest) independently predicted sperm recovery, whereas Sertoli-cell-only and early maturation-arrest patterns did not [77]. In these high-stakes scenarios, complete (bilateral, where indicated) correction of refluxing venous channels is mechanistically rational, though indications remain individualized and guideline support is qualified [4,75].

Recent EAU-panel critical considerations align with that caution: they note possible benefit in selected men with clinically detectable varicocele and NOA, no known genetic abnormality, and a partner without diminished ovarian reserve, but judge the evidence insufficient for a strong recommendation and emphasize counseling about variable outcomes [76].

### 6.5. Is Varicocele a Progressive Disease?

A bilateral conceptualization intersects with the question of progression. The age-related rise in varicocele prevalence—approximately 10% per decade—is consistent with cumulative valvular failure and suggests that venous disease, including its right-sided component, may extend or worsen over time [31]. Longitudinal school-screening data indicate that a proportion of subclinical varicoceles progress to clinical disease over several years, occasionally with testicular hypotrophy. In a 4-year follow-up of boys with school-screening–detected subclinical varicocele, approximately 28% (95% CI 14–45) progressed to clinically detectable disease, one with >20% ipsilateral testicular hypotrophy [78]; and in a 3-year longitudinal study, progression to a clinical varicocele was markedly more frequent among adolescent athletes with subclinical disease than among healthy controls (36% versus 5%), identifying regular strenuous physical activity as an aggravating factor [79,80]. The quality of longitudinal evidence is modest—the key prospective natural-history cohorts largely predate 2015 and most concern the dominant left side—nonetheless, the temporal dimension supports vigilance, since a subclinical right lesion identified today may acquire clinical and functional significance later. This argues for follow-up rather than dismissal of subclinical contralateral disease, while stopping short of mandating early intervention.

### 6.6. Contralateral Evolution After Unilateral Repair: An Untested Hypothesis

Retroperitoneal and pelvic venous communications make postoperative redistribution of venous flow biologically plausible; however, a reliable incidence of de novo or progressive right-sided clinical varicocele after isolated left repair cannot currently be reported. Available comparative studies mainly evaluate reproductive outcomes in men with pre-existing right subclinical reflux and do not establish that left-sided vein ligation causes contralateral progression, and reports of a right varicocele recognized after unsuccessful left repair cannot distinguish true postoperative progression from an initially missed contralateral lesion. The proposed “hemodynamic overload” should therefore be presented as an untested hypothesis rather than a justification for prophylactic bilateral repair. Men with documented right subclinical reflux who undergo left-only repair may reasonably receive clinical follow-up, with repeat Doppler assessment if symptoms develop, right-sided findings progress, or postoperative semen recovery is unsatisfactory.

## 7. Isolated Right Varicocele: A Special Case

Isolated right-sided varicocele—right disease without a left counterpart—is uncommon and merits separate consideration, both because it is the exception that tests the laterality rule and because of its classic association with secondary pathology.

The longstanding teaching holds that an isolated right varicocele, particularly of new onset, non-decompressing in the supine position, or in an older patient, should prompt evaluation for a retroperitoneal or abdominal mass, renal pathology, or a venous anomaly that obstructs or alters right gonadal venous drainage [81]. Documented causes include right-sided IVC and renal venous anomalies (for example, a left-sided IVC with retro-aortic left renal vein and azygos continuation) that redirect or impede right gonadal drainage [81].

The cancer-association component of this teaching has been re-examined. In a large single-institution retrospective cohort, men with right varicocele were significantly older and heavier and underwent cross-sectional imaging far more often than men with left or bilateral disease, yet laterality was not significantly associated with a higher rate of malignancy [10]. A subsequent multi-institutional study of 210 men with right-sided varicocele likewise identified no underlying malignancy, arguing against routine cross-sectional imaging for malignancy screening on the basis of laterality alone [82]. The appropriate inference is nuanced: the data temper the assumption that isolated right varicocele reliably signals occult cancer, while still supporting a low threshold for imaging in atypical presentations—new onset, failure to decompress when supine, rapid progression, or accompanying systemic features. A pragmatic approach is outlined below.

The varicocele should first be characterized by its onset, reducibility in the supine position, grade, and symptoms, and screened for red flags—new or rapidly progressive disease, non-decompression when supine, constitutional symptoms, hematuria, or a palpable abdominal/flank mass. If any red flag is present, cross-sectional abdominal/retroperitoneal imaging should be obtained to evaluate for a mass, renal pathology, or venous anomaly [10,81]; if the right varicocele decompresses normally and no red flag is present, it may be managed as for varicocele generally, with imaging individualized. Underlying IVC/renal anomalies warrant consideration when findings are atypical or recurrent [81].

## 8. Reframing Varicocele: Unilateral Lesion or Bilateral Disease?

The accumulated evidence permits a measured synthesis rather than a categorical verdict.

The case for conceptualizing varicocele as a bilateral disease rests on several observations. (i) Reported laterality is strongly method-dependent: bilateral rates climb monotonically from palpation to ultrasonography to thermography/venography (Section 4) [15,19,20]. (ii) Subclinical right-sided disease accompanies left clinical disease in a substantial proportion of imaged men [15,20,46]. (iii) A clinically unilateral lesion can exert bilateral effects: in men it produces bilateral scrotal temperature elevation [36], contralateral testicular injury itself is demonstrated in animal models [37], and shared oxidative mechanisms provide a plausible bilateral mediator [42]. (iv) Bilateral disease carries a distinct, more severe molecular and clinical phenotype, and varicocele grade does not track dysfunction [19,51,52]. (v) The reproductive logic—fertility requires only one healthy testis—implies, on reproductive grounds, that varicocele-associated infertility reflects a bilateral functional problem [1,11]. (vi) A consistent surgical “dose-effect,” in which bilateral repair outperforms unilateral repair, is what one would expect if both sides commonly contribute [59,60,61].

The case against the strong form of the hypothesis rests on four considerations. (i) The headline thermographic/venographic figures of ~80–84% bilaterality derive largely from a single group in selected populations of patients undergoing intervention and await independent large-scale replication [11,15]. (ii) Ultrasonographic ascertainment lacks a standardized definition, so “bilateral on imaging” is not a fixed quantity [3,23]. (iii) Demonstrating an impalpable right-sided venous abnormality is not the same as demonstrating that it is clinically consequential or that repairing it in isolation changes outcomes—indeed, isolated subclinical repair does not improve pregnancy [70,71]. (iv) Guideline bodies continue to frame intervention around clinical varicocele and do not endorse routine treatment of subclinical or contralateral subclinical disease [4,5].

Varicocele is most accurately described as an asymmetric disorder. Clinical examination identifies the dominant side, usually the left, whereas systematic imaging may reveal contralateral non-palpable reflux in a subset of men; the frequency of such involvement remains uncertain because published studies differ substantially in population selection, examination protocol, definitions, and reference standards. Separately, human thermal data and experimental models support the possibility that a clinically unilateral lesion may have bilateral functional effects. These two concepts—contralateral venous reflux and contralateral functional injury—should not be treated as equivalent.

The clinical implications depend on phenotype. Bilateral palpable varicoceles in an infertile man with abnormal semen parameters differ fundamentally from a clinical left varicocele accompanied only by imaging-detected right reflux: the former may justify bilateral repair under standard guideline criteria, whereas for the latter the evidence is mixed and current guidelines do not support routine treatment of non-palpable disease. The review therefore supports deliberate bilateral clinical assessment and standardized bilateral CDU when imaging is clinically indicated, but not universal imaging, automatic reclassification of imaging-only reflux as clinically significant disease, or prophylactic contralateral surgery.

## 9. Guideline Positions and Practice Variation

Professional guidelines remain appropriately conservative because evidence linking contralateral subclinical reflux to patient-important outcomes remains heterogeneous. The American Urological Association/American Society for Reproductive Medicine (AUA/ASRM) guideline (2020; amended 2024) continues to anchor varicocele repair to palpable varicocele(s), infertility, and abnormal semen parameters, and recommends against varicocelectomy for non-palpable varicoceles detected solely by imaging (Strong Recommendation, Grade C) [4], a position retained in the 2024 amendment [83]. The 2025 EAU male-infertility update likewise emphasizes thorough urological assessment of infertile men and evidence-based treatment selection rather than routine treatment of subclinical disease [84]. More focused recent consensus documents—a dedicated Global Andrology Forum clinical guideline on infertile men with varicocele [85] and a clinical consensus from the Korean Society for Sexual Medicine and Andrology [86]—concentrate on clinical disease while acknowledging persistent uncertainty around subclinical and contralateral management. A recent EAU-panel commentary on varicocele in azoospermia is similarly cautious, acknowledging possible benefit only in selected men with clinical varicocele and NOA while stressing the limited quality and variability of outcome data [76]. A large global practice survey documented wide disagreement across diagnostic criteria, the role of ultrasonography, and management of subclinical varicocele [5]. Thus, laterality and subclinical contralateral management remain evidence gaps rather than settled guideline mandates.

**Figure 2:**
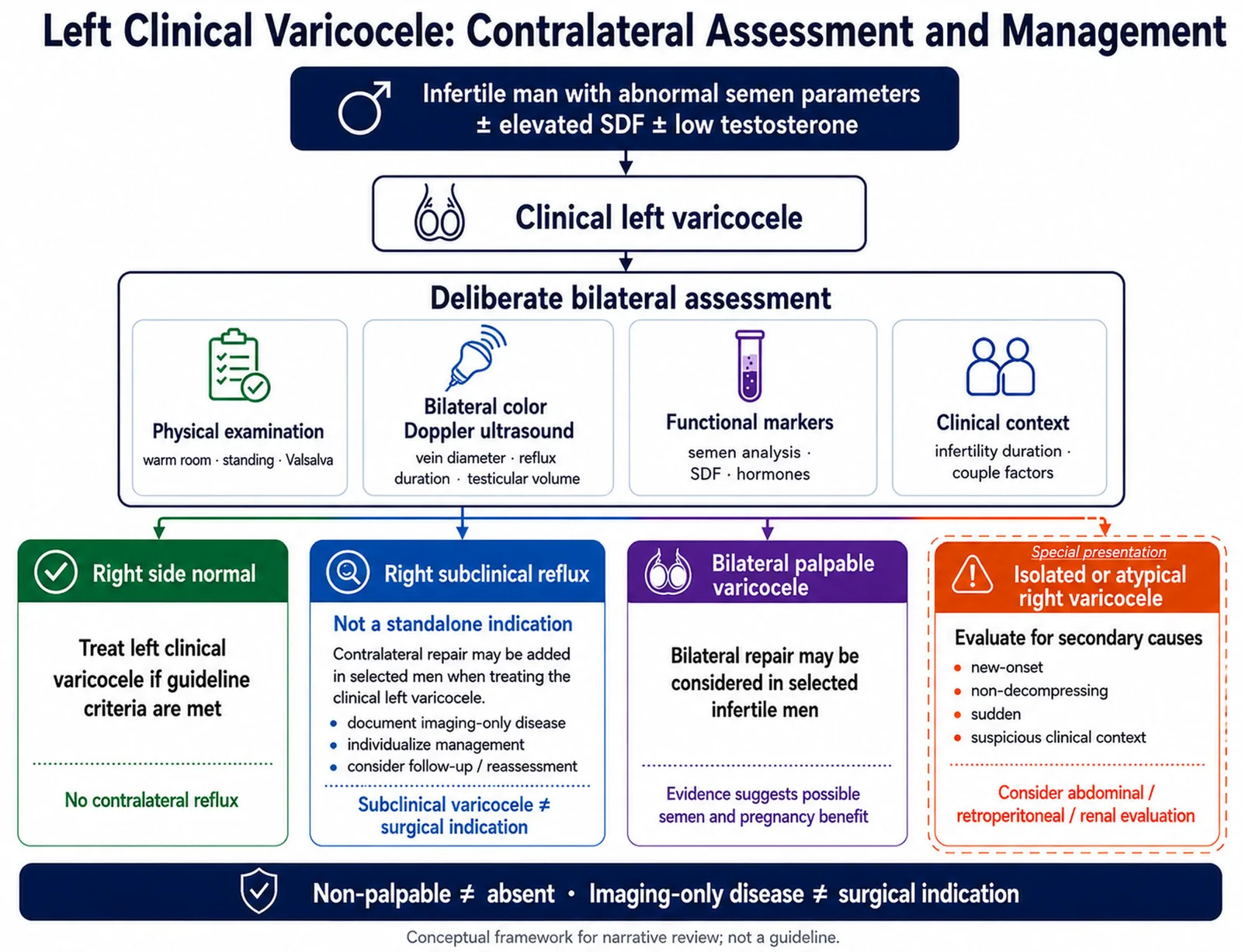
Conceptual framework for contralateral assessment and management in the infertile man with a clinical left varicocele. After a clinical left varicocele is identified in a man with abnormal semen parameters (±elevated sperm DNA fragmentation [SDF] or low testosterone), the contralateral side is evaluated deliberately using physical examination, bilateral color Doppler ultrasonography, functional markers, and clinical context. Management is individualized by contralateral findings: a normal right side warrants treatment of the clinical left varicocele when guideline criteria are met; right subclinical reflux is not a standalone surgical indication, although contralateral repair may be added in selected men when the clinical left varicocele is treated; bilateral palpable disease may warrant bilateral repair in selected infertile men; and an isolated or atypical right varicocele prompts evaluation for secondary causes. The framework reflects the synthesis of the present review and is not a guideline: non-palpable disease is not equivalent to absent disease, and imaging-only disease is not itself a surgical indication.

This guideline position is in explicit tension with the present review’s clinical message, and the tension is worth stating plainly. This review argues for deliberate evaluation of the contralateral side, whereas the AUA/ASRM guideline advises against routine ultrasonography to detect non-palpable disease [4]. The two positions are reconcilable once the aim is specified: guidelines (justifiably) reject population-level ultrasonographic screening to find—and then treat—subclinical varicoceles, because isolated subclinical repair does not improve pregnancy (Section 6.3). The argument advanced here is narrower and is not a call for routine screening or routine contralateral repair; it is that, in a man already undergoing assessment or repair of a clinical varicocele, the contralateral side should not be presumed normal on palpation alone, and its evaluation may inform—rather than dictate—surgical strategy (Figure 2).

Current clinical guidelines distinguish diagnostic characterization from treatment eligibility. The AUA/ASRM and EAU recommend repair for infertile men with a palpable or clinical varicocele and abnormal semen parameters after consideration of the couple’s reproductive context, and advise against treatment of non-palpable lesions detected solely by imaging [4,84]. Radiological recommendations address a different question—once ultrasonography is clinically indicated, it should be performed with a standardized bilateral protocol—so that bilateral imaging may improve characterization and surgical planning without converting an imaging-only right-sided abnormality into an automatic surgical indication [26,27].

## 10. Research Gaps and Future Directions

The central research need is no longer to establish whether varicocele can be bilateral, but to define when contralateral involvement is reproducible, biologically consequential, and treatment-responsive—shifting the field from anatomical laterality toward clinically relevant contralateral involvement.

First, laterality research requires standardized bilateral imaging and reporting: a prespecified color Doppler ultrasonography protocol applied to both sides (patient position, room temperature, Valsalva maneuver, maximum venous diameter, reflux duration, testicular volume, and, where feasible, intratesticular arterial indices), with raw metrics reported rather than binary categories and, ideally, central image review. Without harmonized definitions, bilateral prevalence estimates remain non-comparable across clinical examination, ultrasonography, and venography [22,23,46].

Second, prevalence studies must be separated from treatment-effect studies. Multicenter cohorts should enroll consecutive infertile and non-infertile men, define clinical, subclinical, hemodynamic, and functional bilaterality a priori, and avoid pooling general-population screening with referral-enriched or surgical populations; the very high bilateral rates reported in thermographic, venographic, and expert-reassessment series require independent replication with blinded bilateral assessment and transparent denominators [15,24].

Third, an operational definition of the clinically consequential contralateral varicocele is needed. Rather than a present/absent classification, a right-sided lesion should be phenotyped by reflux burden, testicular-volume asymmetry, intratesticular arterial indices, semen parameters, sperm DNA fragmentation, oxidative-stress markers, reproductive hormones, and emerging sperm proteomic or metabolomic signatures, with prospective models testing which of these predict semen deterioration, endocrine impairment, or benefit from repair [43,45,47,48,51].

Fourth, therapeutic bilaterality should be tested in adequately powered, pragmatic randomized trials. The unresolved population is not isolated subclinical varicocele—which confers limited fertility benefit and does not justify routine surgery—but infertile men already meeting criteria for repair of a clinical left varicocele who also have right subclinical or low-grade reflux. Trials should compare left-only with bilateral repair, stratify by reflux burden and semen/hormonal phenotype, and adopt live birth and time-to-pregnancy as primary endpoints; existing data are directionally favorable but heterogeneous, making patient selection the central issue [61,63,72].

Finally, anatomy-informed surgical mapping should be linked prospectively to reproductive outcomes and recurrence—documenting completeness of bilateral venous control rather than the laterality of the incision—and guideline implementation should be studied directly, evaluating whether deliberate contralateral assessment improves counseling, surgical planning, and recurrence prevention without prompting automatic surgery for imaging-only disease. Non-palpable is not absent, but imaging-only disease is not itself a surgical indication [69,83,84,85].

## 11. Conclusions

Varicocele is traditionally taught as a left-sided, essentially unilateral disease, yet this framing sits in tension with both reproductive logic and the accumulated data. As developed above, the most defensible reading is that varicocele is frequently a bilateral disorder with markedly asymmetric expression—a clinically unilateral presentation of bilateral testicular dysfunction—whereas the strong claim that it is almost universally bilateral rests on selected cohorts and remains a hypothesis pending independent confirmation. For the clinician the message is concrete and immediate: evaluate both sides deliberately, do not equate “non-palpable” with “absent,” individualize the decision to address contralateral subclinical disease, and treat an isolated right-sided varicocele as a presentation warranting anatomical scrutiny. In this respect, varicocele-associated infertility is inherently a problem of personalized medicine: phenotype-based characterization of the contralateral testis—by reflux burden, semen and sperm-DNA-fragmentation profile, and endocrine markers—should guide an individualized repair strategy rather than a uniform, laterality-based approach. Imaging-only right-sided reflux should not automatically trigger surgery, because validated predictive criteria and consistent patient-important benefits remain lacking; the central research priority is to identify reproducible contralateral phenotypes that predict functional deterioration or benefit from repair.

## Figures and Tables

**Table 1:** Characteristics, clinical uses, and limitations of methods used to assess varicocele laterality.

Modality	What It Directly Assesses	Ability to Identify Non-Palpable Reflux	Typical Clinical Setting	Main Limitation
Physical examination (palpation)	Palpable or visible venous dilatation and clinical grade	No	Routine clinical evaluation and determination of clinical varicocele	Operator- and condition-dependent; cannot identify imaging-only disease [3,20]
Color Doppler ultrasonography	Venous diameter, presence and duration of reflux, testicular volume, and reflux anatomy	Yes	Equivocal or difficult examination, documentation of testicular volume, suspected persistence/recurrence, and selected preoperative mapping	Protocol, threshold, and operator variability; may identify abnormalities of uncertain clinical significance [3,23]
Contact thermography	Scrotal surface temperature patterns	Indirectly	Research and selected historical series	Limited independent validation and little contemporary clinical use [15]
Spermatic venography	Refluxing venous channels, duplications, and collateral pathways	Yes	Embolization and selected recurrent or anatomically complex cases	Invasive; strong referral and verification bias; unsuitable for population screening [11,15]

Note: The columns describe the type of information obtained and the usual clinical context of each modality; they are not head-to-head estimates of sensitivity or specificity, because the cited studies involved different populations, ages, operators, protocols, and reference standards.

**Table 3:** Studies comparing bilateral with unilateral varicocelectomy (palpable left with low-grade or subclinical right, or bilateral palpable disease). MA, meta-analysis; OR, odds ratio; NS, not significant.

Study (Ref)	Design	Varicocele Pattern	Key Result (Bilateral Versus Unilateral)
Scherr & Goldstein 1999 [59]	Prospective comparative	Left II–III + right I (palpable)	Motile sperm conc. ≈+96% versus ≈+43% (*p* < 0.05)
Libman et al. 2006 [60]	Comparative cohort	Bilateral palpable	Motility ≈+8% versus ≈+4%; pregnancy 49% versus 36%
Elbendary & Elbadry 2009 [65]	Prospective comparative study (RCT in some meta-analyses)	Left clinical + right subclinical	Greater semen + pregnancy gains with bilateral repair
Sun et al. 2018 [61]	Randomized controlled trial (*n* = 358)	Left clinical + right subclinical	Pregnancy 42.5% versus 26.0%; better conc./morphology/motility
Zheng et al. 2009 [62]	Randomized controlled study (*n* = 104)	Left clinical + right subclinical	No benefit: NS differences in concentration, motility, morphology, testicular volume, testosterone, or pregnancy
Ou et al. 2019 [63]	MA	Bilateral varicocele	Pregnancy OR ≈ 1.9; greater motility gains
Niu et al. 2018 [64]	MA of RCTs	Left clinical + right subclinical	Pregnancy OR ≈ 1.7; concentration NS

Note: Bilateral palpable disease and imaging-only right-sided reflux represent different clinical phenotypes. Definitions of subclinical disease, surgical approaches, follow-up duration, and reported outcomes varied substantially; pooled findings should not be interpreted as a universal indication for contralateral repair. Abbreviations: MA, meta-analysis; RCT, randomized controlled trial; OR, odds ratio; NS, not significant.

## Data Availability

No new data were created or analyzed in this study. Data sharing is not applicable to this article.

## Abbreviations

The following abbreviations are used in this manuscript:

ASRM	American Society for Reproductive Medicine
AUA	American Urological Association
BMI	body mass index
CDU	color Doppler ultrasonography
CI	confidence interval
EAU	European Association of Urology
FSH	follicle-stimulating hormone
ICSI	intracytoplasmic sperm injection
IVC	inferior vena cava
LH	luteinizing hormone
MRI	magnetic resonance imaging
NOA	non-obstructive azoospermia
OR	odds ratio
RCT	randomized controlled trial
ROS	reactive oxygen species
SDF	sperm DNA fragmentation
